# Cardiac Safety of Intranasal Chlorpheniramine: An Exposure-Based Risk Assessment

**DOI:** 10.3390/ph19050670

**Published:** 2026-04-25

**Authors:** César Alas-Pineda, Dennis J. Pavón-Varela, Kristhel Gaitán-Zambrano, Gustavo Ferrer

**Affiliations:** 1 Moxie Health Group, Department of Research & Development, Hallandale Beach, FL 33009, USA; dennisjpavon@gmail.com (D.J.P.-V.); kmelissag13@gmail.com (K.G.-Z.); 2 Department of Pulmonary and Critical Care Medicine, Aventura Hospital and Medical Center, Aventura, FL 33180, USA; gferrer@pulmonary-institute.com

**Keywords:** chlorpheniramine, intranasal antihistamines, cardiotoxicity, QT prolongation, hERG, pharmacokinetics, drug safety

## Abstract

**Background:** H1-antihistamines are widely used for allergic and upper respiratory conditions; however, several agents included in this class have been associated with cardiac electrophysiological adverse effects, including QT interval prolongation and torsades de pointes (TdP). These effects are largely exposure-dependent and mechanistically linked to inhibition of cardiac ion channels. Chlorpheniramine maleate (CPM), a first-generation H1-antihistamine, has been implicated in arrhythmic events primarily under conditions of increased systemic exposure, prompting interest in whether alternative routes of administration may lower cardiac risk. **Methods:** This narrative review integrates mechanistic, preclinical, clinical, pharmacokinetic, and regulatory evidence. Information was extracted from PubMed, Google Scholar, and Scielo using search terms such as cardiotoxicity, chlorpheniramine, QT prolongation, intranasal administration, and cardiac arrhythmias, with no language restriction. **Results:** Comparative pharmacokinetic evidence shows that, on a dose-normalized basis, intranasal and oral chlorpheniramine exhibit comparable bioavailability; however, in a clinical context, intranasal doses (1.12–2.24 mg) are lower than oral daily doses (4–12 mg/day), resulting in a lower systemic exposure (Cmax and AUC) with intranasal administration. Available pharmacovigilance or epidemiological data have not specifically evaluated intranasal chlorpheniramine, and the number of dedicated safety trials remains limited. **Conclusions:** Preclinical, in vitro, mechanistic studies suggest that intranasal administration of chlorpheniramine should confer superior cardiac safety compared to the oral route. However, clinical data from human studies directly comparing the cardiac safety of intranasal chlorpheniramine versus systemic chlorpheniramine is extremely limited. More data from clinical trials, case–control studies, and regulatory databases are needed to validate these theoretical claims.

## 1. Introduction

H1-antihistamines are widely used for allergic and upper respiratory conditions. The H1 receptor is widely expressed across multiple cell types, including epithelial cells, vascular smooth muscle, neurons, glial cells, and immune cells. Upon histamine activation, this receptor activates a cascade of responses culminating in the signs and symptoms of allergic and inflammatory reactions [1]. Notably, in cardiac tissue, histamine employs chronotropic and inotropic effects. Furthermore, recent literature implicates this pathway in the pathogenesis of several forms of cardiac injuries, principally through H1 receptor-mediated coronary vascular reactivity and the development of atherosclerosis [2].

In 1937, antihistamines were introduced. Since then, they have developed considerably, advancing from first-generation compounds associated with central nervous system side effects due to their capacity to cross the blood–brain barrier to second-generation agents designed to avoid these limitations [1]. Despite their extensive use for nearly 90 years, several agents, particularly first-generation compounds, have been associated with adverse cardiac electrophysiological effects, including QT interval prolongation and torsades de pointes (TdP). As a result, drug-induced long QT syndrome (diLQTS) has emerged as a critical safety consideration in drug development and regulatory decision-making, driven largely by a mechanistic understanding of cardiac ion channel blockade, especially inhibition of the rapid delayed rectifier potassium current (I_Kr) mediated by the hERG (KCNH2) channel [3,4,5,6,7]. Epidemiological data suggest an annual incidence of approximately 3.6 Tdp cases per 100,000 persons [8]. Moreover, a higher incidence of cardiotoxicity has been documented in association with second-generation H1 antihistamines, predominantly terfenadine and astemizole [9], with risk increased under critical conditions of overdose, hepatic dysfunction, or pharmacokinetic interactions with CYP450 inhibitors such as azole antifungals, macrolide antibiotics, and imidazole-based compounds [4]. Finally, QT Interval prolongation is an important electrocardiographic finding, as it remains the best marker for predicting the risk of TdP. QT dispersion was used as a marker, but it is not widely used in patients with a history of TdP or QT interval prolongation due to limited evidence that these conditions increase QT dispersion [10].

Chlorpheniramine is a first-generation H1-antihistamine used to manage allergy symptoms. It also triggers eosinophil cell death by activating the JNK pathway, reducing pain signals, lowering oxidative stress by limiting reactive oxygen species, and delaying early viral entry. Due to antimuscarinic effects, chlorpheniramine can increase the cardiovascular response to noradrenaline [11], which may relate to arrhythmias seen in case reports and pharmacovigilance data, mostly with overdose or interactions [11,12,13,14,15,16,17]. These safety issues are mainly linked to oral use, where drug exposure is highest.

There is a growing interest in intranasal administration as an alternative drug delivery route, primarily due to its potential to reduce systemic drug exposure. Unlike oral administration, the intranasal route bypasses first-pass hepatic metabolism, potentially reducing the risk of fatal adverse effects [18]. Additionally, being non-invasive supports patient compliance with treatment [19]. Drug absorption through the nasal epithelium—via paracellular and transcellular pathways in liquid formulations—facilitates drug penetration across otherwise impermeable tight junctions [20,21]. For optimal intranasal absorption, formulations should maintain a pH of 4.5 to 6.5 to prevent nasal irritation and preserve ciliary clearance, as disruptions can lead to local side effects such as dryness, irritation, and nasal itching [20]. The formulation characteristics enable a faster onset of action through efficient mucosal absorption. By using a more targeted route of administration, lower doses are required, which may reduce systemic side effects and, perhaps, improve the risk profile [22].

Although chlorpheniramine has been used for decades and interest in intranasal drug delivery is growing, no review has examined whether intranasal H1 antihistamines are cardiotoxic. Most data on antihistamine-related cardiotoxicity focus on oral formulations and systemic exposure, leaving the lower systemic exposure and potentially reduced cardiac risk of intranasal administration unaddressed. With the introduction of additional intranasal antihistamine products and increased safety concerns, synthesizing mechanistic, preclinical, and clinical evidence has become progressively important.

## 2. Methods

We performed a narrative literature review of current evidence on H1-antihistamines, focusing on chlorpheniramine and its cardiovascular safety profile. Our main objective was to collect data on pharmacokinetics, pharmacodynamics, clinical outcomes, epidemiology, and real-world pharmacovigilance. Searches were conducted in PubMed, Google Scholar, and Scielo. The search included publications from March 2015 to February 2026. Search terms were combined, and the following search strings were used, adjusted for each database: (“Cardiotoxicity/blood”) AND “Chlorpheniramine”) AND “Torsades de Pointes/etiology”) AND “Administration, Intranasal/methods”) AND “Arrhythmias, Cardiac”.

Earlier studies (prior to March 2015) were included only if recent literature lacked essential information. No language restrictions were applied in order to minimize selection bias. The reference list of the included studies was systematically screened to identify additional relevant sources not captured by the initial search strategy. The last search was performed in March 2026.

Studies were included if they reported valuable data on the mechanism of action, mechanism of cardiotoxicity, QT prolongation, arrhythmogenic potential, systemic or intranasal administration, pharmacodynamics, and pharmacokinetics evidence related to H1-antihistamines, particularly chlorpheniramine. Eligible study designs included observational studies, systematic and narrative reviews, crossover studies, case reports, and pharmacovigilance analyses. Key variables of interest included population demographics, administration route, study design, pharmacokinetics (Cmax, AUC, bioavailability), and adverse effects. Studies were excluded if they lacked data on pharmacokinetics, pharmacodynamics, or cardiac safety. No formal quality assessment tool was applied. Methodological limitations of key studies are addressed in the Limitations section.

Studies extracted from each database underwent a two-phase screening process. The first phase consisted of an evaluation of titles and abstracts against established eligibility criteria. In phase two, the full text of potentially relevant articles was assessed for inclusion. Study selection was performed by the authors, and discrepancies were resolved through a consensus.

A total of 44 references aligned with established inclusion criteria. Relevant data were extracted, synthesized, and organized into key subheadings for clarity and coherence, including mechanisms of cardiotoxicity; cardiac safety of chlorpheniramine and H1-antihistamines; pharmacokinetics of systemic and intranasal administration; clinical pharmacodynamic data; and pharmacovigilance from real-world evidence. We compared intranasal and systemic routes of administration to evaluate exposure-dependent effects, with particular emphasis on dosing parameters and cardiovascular events. Results are presented transparently to allow readers to assess the evidence-based limitations of this review.

## 3. Mechanisms of H1-Antihistamine-Induced Cardiotoxicity

Intranasal chlorpheniramine may provide double benefits by combining the mechanism of action of chlorpheniramine with the advantages of an intranasal formulation. CPM inhibits histamine-mediated inflammation and reduces viral adsorption, demonstrating virucidal properties in cases of upper respiratory infections [23].

Based on these therapeutic benefits, it is also important to consider the safety profile of H1-antihistamines. Drug-induced QT prolongation and torsades de pointes have been consistently linked to systemic exposure to H1-antihistamines, particularly in the setting of overdose or pharmacokinetic interactions [4,5,13,15]. The risk of cardiotoxicity is higher in certain populations, including women, individuals with heart disease, those who already have arrhythmias, or those with electrolyte disturbances such as hypokalemia, hypocalcemia, or hypomagnesemia. Use of other medications that block IKr channels may further increase cardiovascular risk by interacting with these antihistamines, thus elevating the chance of ventricular arrhythmias [24].

### 3.1. hERG Channel Blockade and QT Prolongation

Antihistamine-associated QT prolongation occurs mainly through inhibition of the hERG (KCNH2) potassium channel, which mediates the rapid delayed rectifier current (I_Kr). I_Kr is essential for ventricular repolarization [4,7,25,26,27]. Inhibition of I_Kr delays phase 3 repolarization, prolongs the QT interval, and increases susceptibility to early afterdepolarizations that may trigger TdP [3,4].

Extensive electrophysiological and structural analyses have demonstrated that many non-cardiac drugs, including antihistamines, bind within the hERG channel pore, disrupting normal gating and repolarization dynamics [7,25,28]. Reviews by Roden and colleagues affirmed that even minimal reductions in repolarization reserve may precipitate arrhythmia in susceptible individuals [3,5]. Repolarization reserve is the heart’s capacity to maintain normal ventricular repolarization despite a loss of repolarizing currents. It is demonstrated by a shortening of ventricular repolarization at higher heart rates and prolongation at lower heart rates. This inverse relationship acts as a critical buffer in the automatic regulation of ventricular electrical activity [29].

Both first- and second-generation H1-antihistamines demonstrate hERG interactions in vitro. The arrhythmic risk correlates more closely with systemic exposure than with antihistamine “generation” [7,13,25,27]. The withdrawal of terfenadine and astemizole after reports of severe QT prolongation and TdP underscored the central role of exposure and pharmacokinetics in determining risk [6,7].

Chlorpheniramine has weaker hERG blockade than withdrawn agents, but shows concentration-dependent electrophysiological effects, particularly at supratherapeutic levels or with metabolic inhibition [13,14,28]. Salata et al. compared the IC50 values for iKr blockade with chlorpheniramine, astemizole, and terfenadine in guinea pig myocytes and anesthetized dogs. They observed a higher IC50 for chlorpheniramine, which suggests lower iKr blockade and less cardiotoxic potential [13].

### 3.2. Additional Contributors: Sodium Channel Blockade, Antimuscarinic Effects, and Drug–Drug Interactions

While hERG inhibition is central, other mechanisms may contribute to arrhythmic vulnerability. For example, sodium channel blockade, reported for several first-generation H1-antihistamines at high concentrations, may slow depolarization, widen the QRS complex, and impair intracardiac conduction [26,28]. In addition, antimuscarinic effects may alter autonomic tone and pacemaker activity [12]. Furthermore, drug–drug interactions involving cytochrome P450 pathways can elevate plasma concentrations and reduce repolarization reserve, thereby amplifying QT-related risk [3,6,14]. Taken together, these mechanisms often coexist, which explains why clinically significant arrhythmias may occur even in the absence of marked QT prolongation.

### 3.3. Electrophysiological Effects of H1-Antihistamines

Experimental studies have shown that several first-generation H1-antihistamines exert direct electrophysiological effects on cardiac ion channels [25]. Salata et al. demonstrated that astemizole and related antihistamines significantly inhibit cardiac potassium currents, producing marked QT prolongation in vitro [6,13,25]. Subsequent work confirmed that antihistamine-induced QT effects are dose-dependent and closely linked to hERG affinity [6,25,26]. Additionally, chlorpheniramine remains capable of inducing arrhythmogenicity at elevated plasma concentrations or impaired clearance [5,6]. Collectively, these outcomes indicate that cardiovascular risk depends on systemic exposure that affects ion channels.

## 4. Cardiac Safety of Chlorpheniramine and H1-Antihistamines

### Safety Requirements

Drug-induced QT prolongation is a major safety concern in drug development due to its link to increased TdP risk. The FDA and the International Council for Harmonization of Technical Requirements for Pharmaceuticals for Human Use (ICH E14 guideline) establish a threshold of a 10 ms or greater increase as a trigger for extensive ECG monitoring and arrhythmic risk evaluation, especially in patients with other risk factors. The FDA recommends concentration-QT modeling [8], making QT interval prolongation a key parameter in drug development, assessed via concentration-QT modeling for arrhythmogenic risk.

Several H1-antihistamines with documented QT prolongation risk have been identified through clinical, mechanistic, and regulatory evaluation. These agents differ in arrhythmic liability and regulatory outcomes, largely reflecting differences in systemic exposure and ion-channel affinity. Table 1 summarizes select H1-antihistamines with documented QT prolongation risk.

## 5. Pharmacokinetic: Systemic vs. Intranasal Administration

### 5.1. Pharmacokinetic Evidence in Animal Studies

A comprehensive pharmacokinetic review of chlorpheniramine maleate across different species (rodents, rabbits, bovines, canines, and horses) demonstrated moderate oral bioavailability (25–50%), significant first-pass metabolism, extensive tissue distribution, and a prolonged elimination half-life [30].

### 5.2. Oral Pharmacokinetics in Humans

After oral administration, the peak plasma concentration is reached approximately 2.8 h later. The elimination half-life is 27.9 h [31]. Oral chlorpheniramine is typically administered at total daily doses of 4–12 mg. This results in systemic exposure sufficient to engage cardiac ion channels under adverse conditions [3,13,28].

### 5.3. Intranasal Pharmacokinetics in Humans

In a single-dose, three-way crossover study in healthy subjects, chlorphenamine maleate was nasally administered (0.4% nasal spray) at doses of 1.12 mg and 2.24 mg. Following administration, absorption was rapid, with peak plasma levels between 0.25 and 3 h. On a dose-normalized basis, mean peak plasma concentrations (Cmax) for intranasal spray were 1.24 ng/mL (1.12 mg) and 1.21 ng/mL (2.24 mg). For comparison, Cmax after an 8 mg oral tablet was 1.43 ng/mL. Likewise, AUC_0_–∞ values were 26.44 ngxh/mL (1.12 mg) and 25.56 ngxh/mL (2.24 mg) after intranasal administration, while the oral dose yielded 25.91 ngxh/mL. Thus, on a dose-normalized basis, the systemic bioavailability of intranasal and oral CPM is comparable. Since clinically intranasal doses are lower than standard oral doses, the systemic exposure (Cmax and AUC) is substantially lower with intranasal administration [32].

To illustrate the relevance of intranasal antihistamine, olopatadine is a notable example, available as a nasal spray at 0.6% and used twice daily, with each spray delivering 665 μg. In healthy individuals, Cmax is reached in 30 min to 1 h, whereas in patients with seasonal allergic rhinitis (SAR), the time ranges from 15 min to 2 h. The half-life is 8 to 12 h, and elimination occurs mainly via renal clearance [33]. Likewise, intranasal azelastine, a non-sedating H1-antihistamine, reaches Cmax in 2 to 3 h and is primarily metabolized by cytochrome P450 enzymes, such as CYP3A4, CYP2D6, and CYP1A2 [34].

Supporting these findings, a crossover study by van Toor et al. demonstrated that nasal administration results in substantially lower plasma concentrations and overall exposure compared to oral dosing. This reduction in systemic exposure aligns with a potentially decreased risk of systemic adverse effects, including cardiotoxicity [32]. As a result, intranasal administration reduces the likelihood of achieving plasma concentrations associated with cardiovascular effects. Table 2 provides a direct comparison of systemic and intranasal chlorpheniramine exposure.

## 6. Clinical Pharmacodynamic Evidence

### 6.1. Preclinical and Translational Evidence

Preclinical studies have been key in defining the electrophysiological mechanisms underlying drug-induced arrhythmias and in shaping cardiac safety guidelines [5,27]. Electrophysiological studies using expression systems and isolated cardiomyocytes show that many H1-antihistamines interact with cardiac potassium and sodium channels in a concentration-dependent way. hERG channel affinity aligns with QT liability, while sodium channel effects may worsen conduction abnormalities at higher exposures [7,25,26,27]. The main interaction for cardiac safety is potassium and sodium channel exposure to systemic H1-antihistamine concentrations.

Translational frameworks that combine ion channel data, action potential measurements, and in vivo electrophysiology indicate that clinically meaningful arrhythmia risk arises only when systemic concentrations exceed safety margins [5,27]. Supporting this, animal models, such as Langendorff heart preparations, have shown dose-dependent QT prolongation, QRS widening, bradycardia, and ventricular ectopy in response to first-generation H1-antihistamines [5,23,26,30]. In contrast, limited clinical and translational evidence for intranasal chlorpheniramine consistently demonstrates a favorable safety profile [23,26,32], with systemic concentrations within safety margins and no concerning cardiotoxicity.

### 6.2. Clinical Pharmacodynamic Data for CPM

Real-world pharmacovigilance analyses link H1-antihistamines, especially chlorpheniramine, to QT prolongation [17]. Though direct clinical evidence of chlorpheniramine causing arrhythmia is limited, case reports note TdP and cardiac arrest in the context of concomitant medication use, metabolic inhibition, or reduced repolarization reserve, sometimes without baseline QT prolongation [15].

Further supporting the safety profile of intranasal chlorpheniramine, a randomized controlled pilot trial examined 16 patients, aged 18–25, over 30 days with intranasal CPM + Xylitol spray and no oral treatment in the three months prior to the study. To these patients, intranasal chlorpheniramine was administered at a total daily dose of 1.25 mg, which was not associated with QT prolongation or clinically relevant cardiac adverse events; Sanchez-Gonzalez concluded that this route of administration was effective in the management of allergic rhinitis symptoms with a high safety profile based on a small sample of 16 patients [35].

Recent data support the clinical effectiveness and safety of intranasal olopatadine. Phase III trials demonstrated that intranasal olopatadine, particularly in combination with mometasone, meaningfully improves nasal symptom scores compared with placebo and monotherapy, while maintaining a favorable safety profile [36]. A phase I study evaluated the safety and tolerability of intranasal azelastine in 54 patients who received one of three formulations (0.10%, 0.15%, and a commercially available 0.10%). All formulations were well tolerated and demonstrated a high safety profile, with only mild adverse events reported, including rhinorrhea and sneezing [37].

### 6.3. Real-World Evidence for QTc Prolongation with Antihistamines

Real-world pharmacovigilance evidence specifically evaluating QTc prolongation associated with antihistamines remains extremely limited. Kim et al. highlight this gap; however, crucial data can be extracted from their case-crossover study, which showed that 20% of H1-antihistamines increased the risk of cardiovascular events in the pediatric population within 15 days of drug exposure [16].

A recent retrospective study by Giovannoni et al. [38] proposed a statistical method to quantify the probability of QT prolongation following the initiation of QT-prolonging drugs. Using available clinical data from hospitalized patients, a general linear mixed model (GLMM) predicted absolute QT values and drug-induced changes considering baseline QT, demographic characteristics (such as age, although sex is mentioned as a risk factor in older literature, it was not relevant in this study), and concomitant medications. The study demonstrated a modest but prominent association, highlighting the benefit of integrating pharmacokinetic exposure and subject-specific factors in assessing arrhythmogenic risk. Table 3 summarizes available clinical and pharmacodynamic studies evaluating chlorpheniramine’s cardiac safety.

In a cross-sectional study, Rossi et al. evaluated a total of 243 hospitalized elderly patients; 36.6% had long QT syndrome on admission and 89.7% were receiving at least 1 QT-prolonging medication. In total, 30.5% were on a known-risk medication for TdP. Additionally, one-third were receiving more than two QT-prolonging medications. These patients also had concomitant conditions such as hypocalcemia, left ventricular hypertrophy, and hypertension. Rossi et al. described chronic use of a QT-prolonging drug as more than 4 days; guided by this definition, 67.9% were chronic users of at least one of these drugs. In every situation, risk factors should be considered, as the majority of these medications were prescribed in a general practice context [39]. Specific risk factors, polypharmacy, comorbidities, and drug-specific mechanisms of action cumulatively contribute to QT prolongation in clinical practice.

## 7. Epidemiological and Pharmacovigilance Evidence

To date, no epidemiologic or pharmacovigilance analyses regarding intranasal CPM are available. There is a dearth of clinical evidence available at present to substantiate the cardiovascular superiority of intranasal CPM suggested by preclinical animal and mechanistic studies.

Based on this lack of intranasal CPM data, regulatory surveillance and observational studies have consistently documented serious cardiovascular events associated with systemic antihistamine exposure, particularly in overdose scenarios, pediatric misuse, and polypharmacy contexts [3,6,15]. Diphenhydramine has emerged as the most frequently implicated H1-antihistamine in reports of TdP, ventricular arrhythmia, and cardiac arrest [15,40,41].

Chlorpheniramine appears less often in arrhythmia databases, but clinical and forensic reports link it to sudden death, often mediated by sedation-associated trauma in older adults [14,15,42,43]. Similarly, European pharmacovigilance analyses further indicate that QT prolongation and TdP are not limited to first-generation agents, reinforcing the need for exposure-focused risk assessment across antihistamines [15].

Among adverse reactions to first-generation and second-generation antihistamines, terfenadine and astemizole have the highest arrhythmogenic risk; loratadine, ketotifen, and cetirizine carry a lower risk. Within the spectrum of cardiovascular adverse events, arrhythmia emerges as the most frequently documented cardiovascular adverse event [44]. Ali et al. describe that the FAERS reported 21 serious cases of TdP, with two cases resulting in deaths linked to the use of astemizole. In comparison, terfenadine reported 59 TdP cases and ten deaths [17]. Differences in reports reflect individual drug profiles rather than a class effect. Table 4 shows pharmacovigilance findings on H1-antihistamine cardiotoxicity.

## 8. Limitations

The literature on intranasal chlorpheniramine is limited, given recent interest in this route compared to widespread systemic administration data. At present, only one randomized controlled pilot trial with a sample size of 16 has evaluated the cardiac safety of intranasal chlorpheniramine in accordance with the ICH E14 guidelines. To our knowledge, no other clinical trials or large-scale clinical studies have evaluated the safety of intranasal chlorpheniramine. Therefore, there is a dearth of clinical data to substantiate the claim that intranasal chlorpheniramine has superior cardiovascular safety compared to systemic chlorpheniramine formulations. The lack of concentration-QT data for intranasal chlorpheniramine and the evaluation of cardiac safety from pharmacokinetic exposure also constitutes another knowledge gap.

Based on the Cochrane’s Risk-of-Bias 2.0 tool, the randomized controlled trial by Sanchez-Gonzalez et al. 2021 [35] is considered to have some risk of bias. This is because there is no mention of allocation concealment, and there were no a priori sample size or power calculations performed. Additionally, this clinical trial was not registered on ClinicalTrials.gov, nor was there a protocol published online prior to the start of the study. There is no CONSORT flow diagram within the published article, and it is unclear if the RCT adhered to CONSORT guidance. All of these aspects reduce the certainty of the evidence from that small pilot trial.

The pharmacokinetic comparison between oral and intranasal administration is based on a crossover study in healthy adult volunteers. While valuable for future research, this does not reflect drug exposure in other groups, such as elderly people, pediatric patients, those with hepatic impairment, or individuals on CYP2D6 inhibitors.

Furthermore, clinicians may consider real factors such as congestive symptoms, administration technique, mucosal integrity, and patients’ cognitive abilities, as these may influence proper administration and absorption. Based on these considerations, future studies with larger sample sizes should be conducted in specific populations to confirm the cardiac safety profile of intranasal chlorpheniramine.

Additionally, longitudinal large-scale studies need to be done, especially focusing on fatal cardiovascular events like sudden cardiac death and torsades de pointes when intranasal chlorpheniramine is prescribed. Actual data relies on markers, mainly on QT interval prolongation. Intranasal formulations, including concentration, excipients, and delivery devices, may influence absorption and side effects, and these factors should be considered in future studies. Reporting bias occurs when cases of TdP or QT-interval prolongation are underreported, affecting pharmacovigilance databases and making it difficult to establish causal relationships.

Although earlier studies provide valuable data, they were conducted under regulatory standards that do not align with contemporary standards. Additionally, three of the four references dealing with intranasal chlorpheniramine are self-citations from the same author group, and two of the references are narrative reviews—not original research studies. While these studies constitute the only available evidence on this topic, they represent a major limitation, underscoring the need for independent replication.

## 9. Conclusions

H1-antihistamines hold a well-established, concentration-dependent capacity to alter cardiac electrophysiology, a characteristic driven by inhibition of the hERG potassium channel, which plays a critical role in maintaining an adequate ventricular repolarization reserve.

Chlorpheniramine seems to exhibit lower affinity to the arrhythmogenic effect than other antihistamines that were ultimately removed from the market for safety reasons. Under conditions of elevated systemic exposure—whether due to high-dose oral administration, pharmacokinetic interactions, or individual variability in drug metabolism—chlorpheniramine may potentially result in clinically significant cardiac adverse events, underscoring the importance of exposure-limiting strategies in its therapeutic use.

Intranasal administration could be considered a potential therapeutic alternative for patients with a history of prolonged QT interval, for polymedicated individuals with concomitant CYP2D6 inhibitors, for elderly patients, and for patients with special conditions that make it difficult to adhere to multiple daily dose schedules.

Extensive evidence suggests that systemic drug concentration is a key driver of arrhythmic risk from H1 antihistamines. Intranasal chlorpheniramine appears to limit systemic absorption compared to oral forms, potentially enabling effective nasal mucosa concentrations while potentially reducing systemic exposure associated with hERG channel inhibition and QT prolongation. Cardiac safety claims are supported by limited and indirect evidence, as the only direct human data comes from three studies, one of which is a self-cited narrative review. The absence of concentration-QT studies for intranasal use prevents definitive conclusions regarding cardiac safety.

Based on available mechanistic and pharmacokinetic data, intranasal chlorpheniramine could represent a potential option in patients with risk factors for QT prolongation, as the intranasal route has been shown to produce lower systemic exposure than oral administration. Nevertheless, these findings are based on the limited available pharmacokinetics studies. We acknowledge this as a potential source of interpretation bias and sought to mitigate its impact through transparent reporting of methodology, data sources, and limitations. Future studies should align with ICH E14 guidelines, report pharmacokinetics and pharmacodynamics findings, and recruit a larger patient sample, including those at risk.

## Figures and Tables

**Table 1:** Select H1-Antihistamines with Documented QT Prolongation Risk.

**Drug**	**Generation**	**QT Risk**	**Regulatory Status**
Terfenadine [4,7,15]	2nd	High	Withdrawn (QT-TdP risk)
Astemizole [4,7,15]	2nd	High	Withdrawn (QT-TdP risk)
Diphenhydramine [15,17]	1st	Moderate-High	OTC; QT warning in overdose
Chlorpheniramine [15]	1st	Moderate	Marketed; QT risk at high doses
Loratadine [15,17]	2nd	Low	Marketed; QT risk at high doses
Cetirizine [15]	2nd	Low	Marketed; generally safe profile

Abbreviations: QT, QT interval; TdP, Torsade de pointes; OTC, over the counter. Note: QT risk classification reflects reported clinical, mechanistic, and pharmacovigilance evidence associated with therapeutic or supratherapeutic systemic exposure. The likelihood of QT prolongation and TdP may be influenced by dose, route of administration, metabolic inhibition, drug–drug interactions, and patient-specific risk factors. Regulatory status reflects historical actions and current labeling at the time of writing. Data derived from mechanistic studies, pharmacovigilance analyses, and regulatory reports [4,7,12,15,17].

**Table 2:** Systemic and Intranasal Chlorpheniramine Exposure

**Study**	**Type of Study**	**Sample Size**	**Route**	**Dose**	**PK Findings**
Van Toor et al., 2001 [32]	Randomized crossover study	24	Intranasal (0.4% CPM)	1.12 mg and 2.24 mg	Dose-normalized basis: Cmax: 1.21–1.24 ng/mL. AUC: 25.56–26.44 ngxh/mL
Alas-Pineda et al., 2025 [30]	Scoping review	44	Oral	4 mg	Cmax: 25.9–32.5 ng/mL. AUC: 8.37–1202 ngxh/mL
Sanchez-Gonzalez et al., 2021 [35]	Randomized double-blind pilot	16	Intranasal	1.25 mg twice a day	Systemic levels lower than 1 mg

Abbreviations: CPM, chlorpheniramine maleate; PK, pharmacokinetics; AUC, area under the concentration-time curve; Cmax, maximum plasma concentration.

**Table 3:** Real-world evidence of cardiac safety of antihistamines.

**Study**	**Study Type**	**Sample Size**	**Drugs**	**Population**	**Findings**
Sanchez-Gonzalez et al., 2021 [35]	Randomized double-blind pilot	16	Intranasal CPM + Xylitol	AR patients	No cardiac events or adverse effects were reported
Giovannoni et al., 2024 [38]	Retrospective study	107	QTc prolonging medications	Hospitalized patients with QTc prolongation	Arrhythmogenic risk should be addressed in polypharmacy
Kim et al., 2023 [16]	Case crossover study	1992	First-generation antihistamines	Pediatric patients	H1-antihistamines are associated with cardiac events within the first 15 days of treatment

Abbreviations: AR, allergic rhinitis.

**Table 4:** Pharmacovigilance Evidence on Cardiotoxicity.

**Study**	**Institution**	**Findings**
Ali et al., 2021 [17]	FAERS	CPM Blocks hERG K+ channel and prolongs QT, producing a higher TdP risk.
Poluzzi et al., 2015 [15]	FAERS/Europe	Dexchlorpheniramine is considered a weaker arrhythmia signal and is associated with SCD, CA, fatVT, and non-fatVT.
Routledge et al., 1999 [6]	UK ADROIT	CPM cardiovascular reactions represented 9.7% of total cases. One CA was reported.

Abbreviations: FAERS, Food and Drug Administration Adverse Event Reporting System; CPM, chlorpheniramine maleate; SCD, sudden cardiac death; CA, cardiac arrest; fatVT, fatal ventricular tachycardia; non-fatVT, non-fatal ventricular tachycardia.

## Data Availability

No new data were created or analyzed in this study. Data sharing is not applicable to this article.

## Abbreviations

The following abbreviations are used in this manuscript:

AUC	Area Under the Concentration–Time Curve
CPM	Chlorpheniramine Maleate
DiLOTS	Drug-Induced Long QT Syndrome
ECG	Electrocardiogram
hERG	Human Ether-à-go-go-Related Gene
iCPM	Intranasal Chlorpheniramine Maleate
Ikr	Rapid Delayed Rectifier Potassium Current
OTC	Over-the-Counter
PK	Pharmacokinetics
QT	QT Interval
TdP	Torsades de Pointes
FAERS	Food and Drug Administration Adverse Event Reporting System
SCD	Sudden Cardiac Death
CA	Cardiac Arrest
fatVT	Fatal Ventricular Tachycardia
non-fatVT	Non-Fatal Ventricular Tachycardia
AR	Allergic Rhinitis
RCT	Randomized Controlled Trial
